# Mental health of individuals at increased suicide risk after hospital discharge and initial findings on the usefulness of a suicide prevention project in Central Switzerland

**DOI:** 10.3389/fpsyt.2024.1432336

**Published:** 2024-09-13

**Authors:** Sophia Werdin, Günther Fink, Sarah Rajkumar, Michael Durrer, Caroline Gurtner, Gregor Harbauer, Ingeborg Warnke, Kaspar Wyss

**Affiliations:** ^1^ Swiss Tropical and Public Health Institute, Allschwil, Switzerland; ^2^ University of Basel, Basel, Switzerland; ^3^ Lucerne Psychiatry, St. Urban, Switzerland; ^4^ Bern University of Applied Sciences, Bern, Switzerland; ^5^ Private Clinic Hohenegg, Meilen, Switzerland

**Keywords:** mental health, suicidal ideation, suicide prevention, self-management, program evaluation, health services research, cross-sectional studies, Switzerland

## Abstract

**Background:**

Supporting individuals in managing their suicidality can prevent suicidal behavior. This study evaluated the suicide prevention project SERO, which was launched in Central Switzerland in 2021. SERO comprises four components: the suicide risk assessment technique PRISM-S, a personal safety plan, mental health first aid courses for relatives, and a self-management app. We assessed the mental health of individuals at increased suicide risk after hospital discharge and evaluated the usage and usefulness of SERO components.

**Methods:**

A cross-sectional study targeted former patients of Lucerne Psychiatry with an increased suicide risk. Between March 2023 and March 2024, we collected data from 24 individuals through a questionnaire administered six months post-discharge. Descriptive statistics characterized sociodemographics, assessed self-efficacy, self-management, and health literacy, and analyzed the usage and usefulness of SERO components. Associations between the usage of SERO components and mental health outcomes were investigated using Wilcoxon rank sum tests.

**Results:**

Mental health assessments indicated, on average, low to moderate levels of self-efficacy, self-management, and health literacy, with substantial variations across individuals. Participants’ exposure to SERO components varied: 83% used PRISM-S for suicide risk assessment, 67% developed a personal safety plan, 38% used the SERO app, and 8% reported that their relatives participated in a mental health first aid course. 50% of safety plan users and 44% of SERO app users found the tools helpful before or during a suicidal crisis. 78% of SERO app users would recommend the app to others.

**Conclusion:**

Low to moderate levels of self-efficacy, self-management, and health literacy underscore the need for targeted interventions to support individuals at suicide risk. Positive feedback on the personal safety plan and the SERO app suggests their potential effectiveness in helping individuals manage their suicidality. Therefore, integrating structured measures for promoting self-management into standard care protocols in psychiatric hospitals and into patients’ lives may contribute to preventing suicides. The main limitation of our study is its small sample size. Future larger-scale studies should investigate user experiences in detail, assess the causal effects of SERO components on specific mental health and suicide outcomes, and evaluate the cost-effectiveness of each component separately and in combination.

## 1 Introduction

Suicide is a significant public health problem that caused 958 deaths in Switzerland in 2022 (1). Suicidality is commonly linked to a psychiatric illness, with major depression being the most prevalent underlying disorder (2). Some individuals at suicide risk are treated in a psychiatric hospital, for example, after a suicide attempt. For people prone to suicidal ideation and behavior, the first months after discharge from a psychiatric facility are marked by a significantly increased suicide risk (3). Similarly, individuals experiencing suicidal ideation and those who have attempted suicide are more likely to be readmitted to the hospital (4). This situation is exacerbated by the fact that many at-risk individuals may not attend the recommended outpatient treatment after hospital discharge, quickly drop out of outpatient therapy, or face substantial waiting times for outpatient consultations and support (5). These and many other challenges highlight the need for targeted and practical suicide prevention (SP) measures to support patients during the vulnerable transition period from inpatient to outpatient care and beyond.

Since March 2021, the project SERO (*Suizidprävention: Einheitlich Regional Organisiert*, in English: suicide prevention: uniformly regionally organized), funded by the foundation *Health Promotion Switzerland*, has been gradually implemented at *Lucerne Psychiatry (lups)* in Central Switzerland. Targeting individuals at increased suicide risk, SERO aims to reduce the number of suicides, suicide attempts, and related (re)hospitalizations in the long-term (6). The focus is on strengthening the self-management of at-risk individuals and support mechanisms for their relatives, alongside promoting coordinated and integrated care by professionals (6). The project organization of SERO has a trialogical structure, involving professionals, individuals with suicidal experience, and relatives. Additionally, the project was developed in close collaboration with individuals at suicide risk and their relatives to create user-friendly solutions (6–8).

SERO is closely aligned with the recommendations of the Swiss Federal Office of Public Health for managing the transition from inpatient to outpatient care for those at suicide risk (9), and comprises four project components that can be used either combined or separately. The first component is the introduction of the Pictorial Representation of Illness and Self-Measurement - Suicidality (PRISM-S) method into the *lups* wards. PRISM-S is a standardized visual technique designed for collaborative suicide risk assessment of the patient and the professional (6, 10). PRISM-S is scientifically validated (11) and has been used for many years, for example, in psychiatric hospitals and psychotherapeutic practice. The second component is the development and use of a personal safety plan that outlines individual coping resources and strategies for managing suicidal crises and aims to prepare those at risk for future incidents (5, 6, 12). Patients compile the safety plan with the support of a professional to ensure that it is realistic and implementable during a crisis situation (13). As a third component, relatives of individuals at suicide risk and other interested persons receive vouchers to participate in mental health first aid courses for talking about suicidal thoughts, so-called ensa courses, at a reduced cost through SERO (6). These courses are offered by the Swiss foundation *Pro Mente Sana* and are designed to equip participants with the skills to support people experiencing a suicidal crisis (14). The fourth SERO component is a newly developed digital self-management app that aims to empower individuals at suicide risk and their relatives to take initial steps for SP at any time (6, 7). The app incorporates digital versions of the PRISM-S method for suicide risk self-assessment and the personal safety plan, complemented by quick access to personal and professional emergency contacts (6). Through these measures, SERO aims to support individuals at suicide risk during the vulnerable transition from inpatient to outpatient care and beyond.

Intermediate outcome measures, such as attitudes towards help-seeking and coping skills, can provide insights into the effectiveness of specific SP measures aimed at individuals at suicide risk (15). These measures serve as proximal effect indicators and are directly linked to the objectives and content of an intervention (16). Key intermediate outcome measures relevant to SP include self-efficacy, self-management, and health literacy. An individual’s self-efficacy is defined as the belief in one’s ability to organize and execute actions needed to achieve specific goals (17). With regard to suicidality, self-efficacy reflects an individual’s perceived capability to maintain their own safety when experiencing impulses to suicidal behavior (18). High self-efficacy can prevent the progression from suicidal ideation to suicidal behavior by promoting the use of effective coping mechanisms (19). This can be particularly important in so-called high-risk situations, such as during drug or alcohol use, under negative emotional states, in physical pain, or when facing interpersonal issues. Individuals experiencing suicidal ideation or those with a history of suicide attempts tend to have lower self-efficacy compared to those without suicidal thoughts (19).

Self-management describes an individual’s capacity, including the knowledge, skills, and confidence, to manage their own health and care (20). A high level of self-management facilitates the recovery from mental illness (21) and is considered a protective factor for suicidality (22). Enhancing individuals’ self-management skills through targeted interventions can reduce symptoms of distress (22, 23), improve mental health-related quality of life, patient activation, and overall psychiatric symptoms (24).

Health literacy encompasses the knowledge, competence, and motivation to access, understand, evaluate, and apply information to tasks related to decision-making on health topics (25, 26). Low levels of health literacy have been associated with, for example, increased hospitalizations, greater use of emergency care, and reduced utilization of preventive services (27). General health literacy is closely related to mental health literacy, which is an important determinant of mental health and could contribute to reducing suicidality (28). For example, poor mental health literacy is one of the most common barriers to help-seeking in cases of mental health problems (29).

Previous research has enhanced our understanding of suicidality and its associated risk factors (30). However, there remains a notable gap concerning effective strategies to support individuals at suicide risk during the transition period from inpatient to outpatient care. This study is the first scientific evaluation of the Swiss SP project SERO, which is designed to address this critical transition, improve protective mental health skills, and prevent suicidal behavior in the long-term.

Our research aimed to assess the self-efficacy, self-management, and health literacy of individuals at increased suicide risk six months after hospital discharge. Additionally, we evaluated the usage of the SERO project components and the usefulness of the personal safety plan and the SERO app in supporting at-risk individuals during the critical post-discharge period.

## 2 Methods

### 2.1 Study design and setting

We conducted a cross-sectional study among patients discharged from *lups*. *Lups* is a psychiatric hospital providing services across inpatient, intermediate, and outpatient care sectors to people of all ages in the cantons of Lucerne, Obwalden, and Nidwalden in Central Switzerland (31). *Lups* employs more than 1,360 staff members and has 317 beds in adult psychiatry (32). In 2022, the cantonal hospital managed for adults about 112,500 care days in its inpatient clinics, 14,000 care days in community-integrated acute care, treated more than 630 cases in day clinics, and 6,850 outpatient cases (32).

### 2.2 Study participants and recruitment

Our study targeted individuals assessed as being at high suicide risk during their treatment at *lups*, and who were discharged within the one-year period between September 2022 and August 2023. We excluded data from patients aged under 18 years and over 65 years. Study participants had to be able to complete a written informed consent form and a questionnaire in German. We identified eligible individuals from patient datasets provided by *lups*, using the inclusion criteria regarding suicide risk, discharge date, and age. The *lups*’ clinical information system records suicide risk assessments from different time points (at admission, during treatment, at discharge). Given the typically fluctuating nature of suicidality (33), the specific timing of suicide risk assessment was not deemed relevant for participant selection.

Thus, we approached all eligible individuals for whom a high suicide risk was documented at any time point during their *lups* treatment. Participant recruitment was conducted through postal mail. Six months after hospital discharge, in the period between March 2023 and March 2024, we sent the study documents, including study information, consent form, questionnaire, and a pre-stamped return envelope, to all eligible individuals. After four weeks, we sent a postal reminder along with all relevant documents to all non-responders. The study documents were sent out by *lups*.

Due to the typically high rehospitalization rate for individuals at suicide risk (34), many patient IDs appeared multiple times in the dataset (with different case IDs and discharge dates). Eligible individuals were contacted only once at the earliest time point within the period covered by the provided patient datasets and were not re-contacted in case of subsequent discharges from *lups*.

### 2.3 Data collection

Data were collected using a questionnaire that could be completed by the participants either digitally via a QR code or on paper. While the digitally submitted questionnaires were directly stored in the survey management system evasys version 9.0, we manually entered the responses from the paper-based questionnaires into evasys.

The survey assessed three mental health endpoints: self-efficacy, self-management, and health literacy, employing the following validated instruments in German: the General Self-Efficacy Scale (GSE) (35), the Self-Management Self-Test (SMST) (36), and the Swiss version of the Health Literacy Questionnaire (HLS19-Q12-CH) (25).

The GSE measures a general sense of self-efficacy through ten items, which are rated on a four-point Likert scale (not at all true, hardly true, moderately true, exactly true; 1 to 4 points). Participants are asked to assess, for example, how well they are able to solve personal problems, to deal with unexpected events, and to recover from setbacks (35). The final composite score with a possible range from 10 to 40 points is calculated by summing up the points of the responses to all ten items. The higher the score, the greater is the individuals’ generalized self-efficacy (35). The GSE has been widely used and validated across diverse cultural contexts and is recognized for its high reliability, with Cronbach’s alpha coefficients typically exceeding 0.80 (37).

The SMST assesses the self-management competence of the study participants and consists of five items. Participants are invited to indicate, for example, how they currently cope with social contacts, set priorities, and make decisions (36). The assessment is done on a five-point Likert scale (very bad, quite bad, moderate, quite good, very good; 0 to 4 points). Again, the total score with a possible range from 0 to 20 points is calculated by summing up the points of the responses to all five items. The higher the score, the greater is the individuals’ self-management competence. The possible scores are categorized into five increments, ranging from very poor self-management (0-4 points) to excellent self-management (17-20 points) (36). The SMST demonstrated strong convergent validity, evidenced by significant correlations with other stress-related psychometric instruments, high internal consistency (Cronbach’s α=0.80), the ability to differentiate between clinical and nonclinical samples, and moderate test-retest reliability (r=0.71) (36).

The HLS19-Q12-CH is a measure of general health literacy developed by the World Health Organization Action Network on Measuring Population and Organizational Health Literacy (M-POHL). Participants are asked to indicate how easy it is for them to understand information about what to do in a medical emergency or to find information on how to handle mental health problems, for example (25). The instrument comprises 12 items assessed on a four-point Likert scale (very difficult, difficult, easy, very easy; 1 to 4 points). The HLS19-Q12-CH score is calculated as the percentage of items that a participant answered with ‘very easy’ or ‘easy’. The higher the score, the greater is the individuals’ general health literacy (25). The HLS19-Q12 demonstrated acceptable psychometric properties and validity across different languages, country contexts, and data collection methods, with the Swiss version (HLS19-Q12-CH) achieving a Cronbach’s alpha of 0.72 (38).

Additionally, we collected information on the usage of and experiences with SERO components, alongside sociodemographic data. Questions related to SERO were developed in collaboration with the project’s initiators, focusing on the usage of the project components and the usefulness of the personal safety plan and the SERO app. The sociodemographic items were designed to align with those in the Swiss Health Survey (39). We added the response option ‘I do not want to answer this question’ to all items to mitigate potential discomfort for participants. The collected data were coded via participant IDs. The age in years was derived from the coded patient datasets provided by *lups*.

### 2.4 Data analysis

We performed descriptive analyses to characterize the sociodemographic profile of the study participants, their scores on the GSE, SMST, and HLS19-Q12-CH, as well as the usage and usefulness of SERO components. To investigate the association between the development of a personal safety plan and the usage of the SERO app with the three mental health outcomes, we employed Wilcoxon rank sum tests with continuity correction. We chose the Wilcoxon rank sum test for its ability to compare median scores between two independent groups (e.g., SERO app users and non-users) without necessitating the assumption of normal distribution (40). This non-parametric test is appropriate for our analysis due to its applicability to small sample sizes and its suitability for ordinal data (40).

Missing data occurred when participants refused to answer certain questions on the questionnaire. For the mental health endpoints, the response ‘I do not want to answer the question’ was also counted as missing. With ≤10% missing responses across the mental health variables, we opted for a full case analysis. Additionally, we performed sensitivity analyses on the three mental health outcomes to evaluate the potential impact of missing data. In these analyses, missing values for each variable were imputed with the mean of the available, non-missing values for that variable, operating under the assumption that the data were missing completely at random. Data analysis was performed using the software R version 4.3.3.

### 2.5 Ethics

Participation was voluntary. The participants received detailed study information via postal mail. Written, informed consent was obtained from all participants. There was no compensation for study participation. Ethical approval was granted by the Ethics Committee of Northwestern and Central Switzerland (ID 2022-00870). The reporting in this manuscript was guided by the Strengthening the Reporting of Observational Studies in Epidemiology (STROBE) Statement for cross-sectional studies (41).

## 3 Results

### 3.1 Selection and recruitment process

We identified 274 (9%) individuals at high suicide risk out of 2,994 patients who were discharged from *lups* over the one-year period between September 2022 and August 2023. Of these people at increased suicide risk, 67 (24%) individuals were excluded because they were aged under 18 years at the time of the participation request. Another 28 (10%) individuals were excluded because they were aged over 65 years. Six months after their hospital discharge, we sent study documents to 179 individuals who met the inclusion criteria. For 28 (16%) of the eligible individuals, the postal mail was undeliverable and returned, for example, if the residential address was no longer valid.

**Figure 1:**
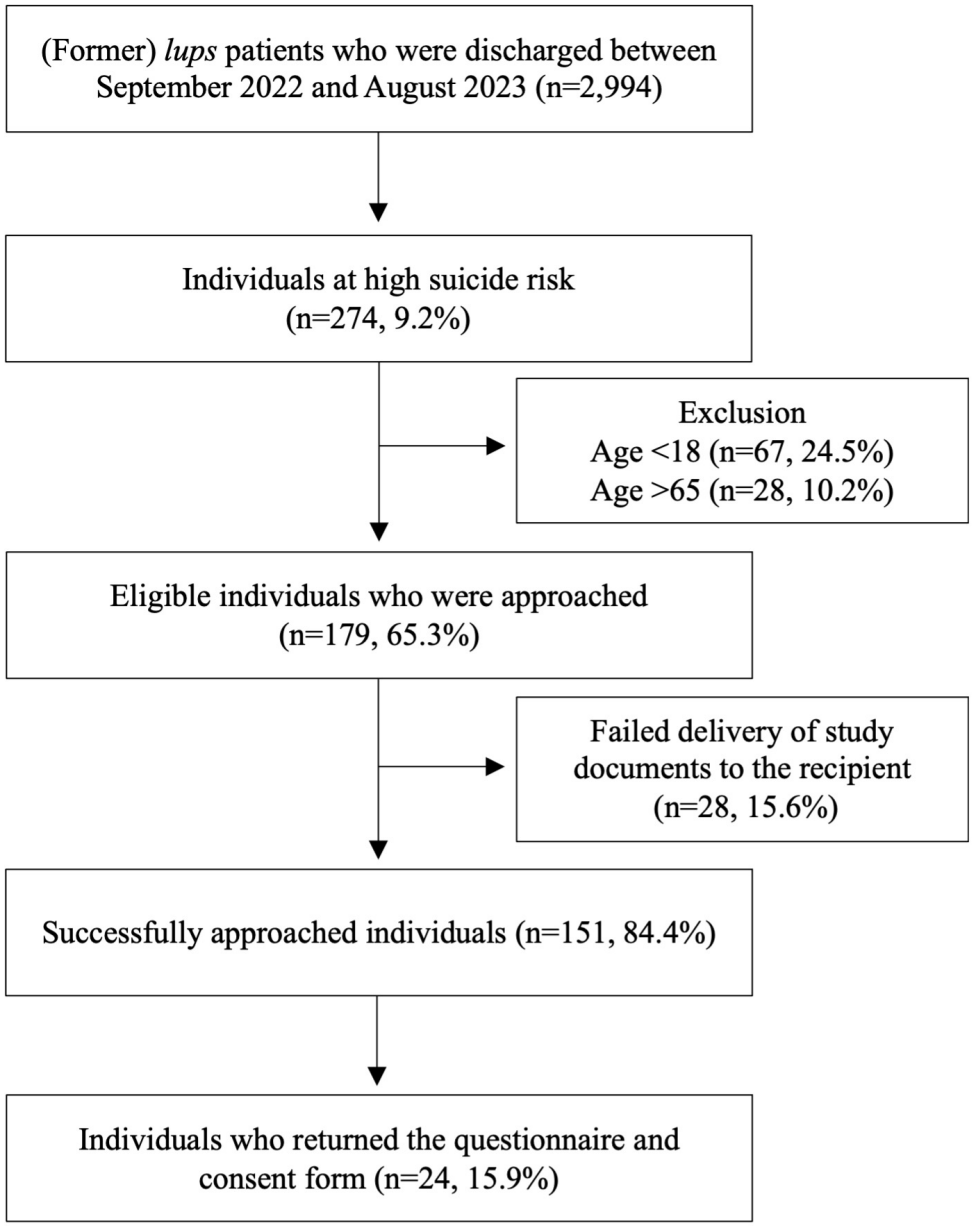
Flow diagram for the selection and recruitment process in our study.

In total, we approached 151 people. Of the successfully contacted individuals, 24 (16%) returned the completed questionnaire and written consent form to *lups*. **Figure 1** displays the flow diagram illustrating the selection and recruitment process in our study. 19 (79%) persons responded after the initial request and 5 (21%) after the reminder. Almost all participants (96%, n=23) completed the questionnaire on paper. Our sample consisted of 20 (83%) female and 4 (17%) male participants, with a mean age of 34.4 years (SD=12.9 years). The sociodemographic characteristics of the study sample are summarized in **Table 1**.

**Table 1:** Sociodemographic characteristics of the study sample (N=24).

Variables	n (%)
Gender	
male	4 (16.7)
female	20 (83.3)
Age in years [mean (SD)]	34.4 (12.9)
Marital status	
single	18 (75.0)
married	2 (8.3)
divorced	3 (12.5)
did not want to answer the question	1 (4.2)
Relationship status	
in a relationship	8 (33.3)
not in a relationship	14 (58.3)
did not want to answer the question	2 (8.3)
Children	
yes	6 (25.0)
no	18 (75.0)
Living alone	
yes	7 (29.2)
no	16 (66.7)
*missing*	*1 (4.2)*
Swiss nationality	
yes	19 (79.2)
no	5 (20.8)
Level of education*	
low	9 (37.5)
medium	12 (50.0)
high	3 (12.5)
Employment status	
employed	6 (25.0)
unemployed or homemaker	5 (20.8)
in training	2 (8.3)
disabled or partially disabled	5 (20.8)
other status	3 (12.5)
did not want to answer the question	2 (8.3)
missing	1 (4.2)
Monthly gross income	
less than 4,000 CHF	12 (50.0)
4,000-5,999 CHF	5 (20.8)
6,000-7,999 CHF	2 (8.3)
did not want to answer the question	4 (16.7)
missing	1 (4.2)

*low = primary/secondary level 1 (compulsory education); medium = secondary level 2 (vocational education and training, and general education); high = professional and higher education (university degree or equivalent). CHF - Swiss francs, SD - Standard deviation.

### 3.2 Self-efficacy, self-management, and health literacy

Participants with complete GSE data (n=21) achieved a mean total score of 22.9 (SD=7.8) on the GSE, with scores ranging from 10 (representing the lowest possible self-efficacy) to 40 points (representing the highest possible self-efficacy). In the sensitivity analysis, by imputing missing values for each variable with the mean of the non-missing values for that variable, participants (n=24) achieved a mean total score of 22.7 (SD=7.4) on the GSE.

Study participants with complete SMST data (n=22) achieved a mean total score of 9.4 (SD=4.2) in the SMST, indicating a moderate self-management competence, with a range from 2 to 15 points (very poor to good self-management competence). According to the categorization of the instrument’s score, none of the individuals in our sample demonstrated excellent self-management competence (17-20 points). 27% (n=6) showed good (13-16 points); 23% (n=5) moderate (9-12 points); 41% (n=9) rather poor (5-8 points); and 9% (n=2) very poor (0-4 points) self-management competence. In a sensitivity analysis that used mean imputation, study participants (n=24) achieved a mean total score of 9.3 (SD=4.0) in the SMST.

Study participants with complete HLS19-Q12-CH data (n=20) achieved a mean total score of 61% (SD=21%) in the HLS19-Q12-CH, with a range from 25 to 100%. According to the categorization of the instrument’s score, none of the participants demonstrated excellent general health literacy (≥50% ‘very easy’ and <8.3% ‘difficult’ and ‘very difficult’ responses). 20% (n=4) showed sufficient general health literacy (>83.3% ‘very easy’ and ‘easy’ responses). Around two-thirds (65%, n=13) of the participants demonstrated problematic general health literacy, defined as all respondents who are not in the groups ‘excellent’, ‘sufficient’, or ‘inadequate’. 15% (n=3) had inadequate general health literacy (<8.3% ‘very easy’ and ≥50% ‘difficult’ and ‘very difficult’ responses). In the sensitivity analysis using mean imputation (treating imputed values ≥2.5 as 3 [‘easy’-responses]), study participants (n=24) achieved a mean total score of 63% (SD=22%) in the HLS19-Q12-CH.

### 3.3 Usage and usefulness of SERO project components

Among the participants, 83% (n=20) indicated that their suicide risk had previously been assessed using PRISM-S. Two individuals (8%) reported that their relatives participated in an ensa course.

In total, 67% (n=16) developed a personal safety plan as part of their treatment at *lups*. Half of these individuals (50%, n=8) indicated that they felt that the personal safety plan could help, or had already helped, before or during a suicidal crisis. 38% (n=6) considered the safety plan not helpful, while 13% (n=2) were unsure. 63% (n=10) of individuals who developed a personal safety plan used it at least once in the 30 days prior to the time of data collection, with two individuals having used it more than ten times. Six individuals used the paper version of the safety plan, four used the tool in the SERO app, and three used both the paper and digital versions. There were two missing values for this variable, and one person declined to answer the question.

**Figure 2:**
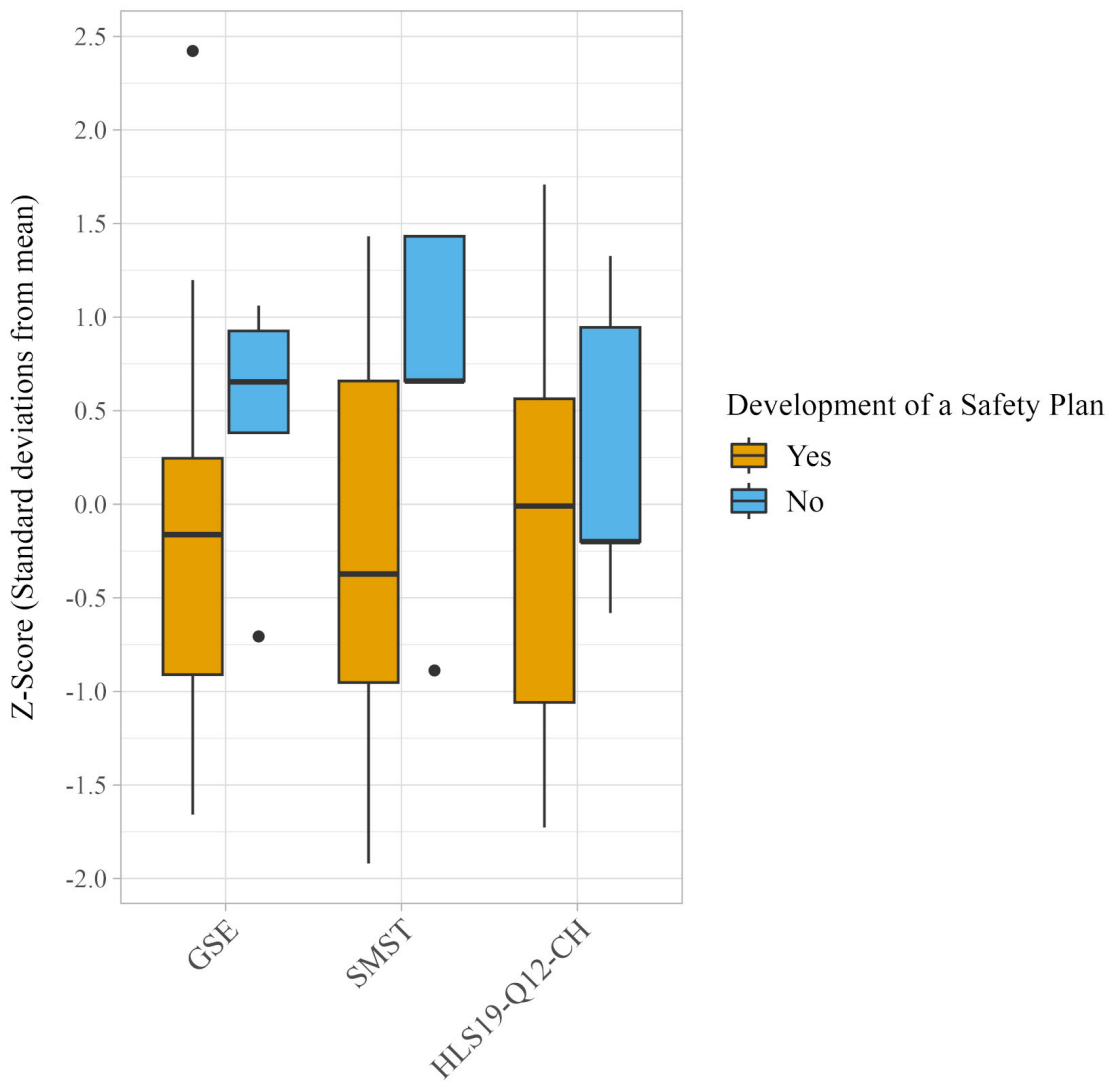
Boxplots depicting the distribution of z-scores on the General Self-Efficacy Scale (GSE), the Self-Management Self-Test (SMST), and the Swiss version of the Health Literacy Questionnaire (HLS19-Q12-CH) categorized by individuals who did and did not develop a safety plan. Z-scores represent the number of standard deviations each participant’s score is from the mean score of the group. Higher scores imply better outcomes. No group difference reached statistical significance (GSE: W=22, p=0.148; SMST: W=20.5, p=0.114; HLS19-Q12-CH: W=34.5, p=0.677).

On average, individuals who did not develop a safety plan reported higher mean scores across all mental health measures compared to those who did. GSE scores averaged 25.6 (SD=5.2) for non-users versus 21.1 (SD=7.7) for users. SMST scores averaged 12.0 (SD=3.7) for non-users versus 8.6 (SD=3.7) for users. HLS19-Q12-CH scores averaged 68.3 (SD=18.1) for non-users versus 60.9 (SD=23.1) for users. However, none of these differences were statistically significant (GSE: W=22, p=0.148; SMST: W=20.5, p=0.114; HLS19-Q12-CH: W=34.5, p=0.677). **Figure 2** presents a comparative boxplot visualization of mental health outcome scores, standardized as z-scores, between participants who did and did not develop a safety plan.

Of the study participants, 38% (n=9) had used the SERO app at least once. Among the app users, 44% (n=4) felt that the SERO app could help, or had already helped, before or during a suicidal crisis. 33% (n=3) considered the SERO app not helpful, while 22% (n=2) were unsure. All individuals who used the SERO app (n=9, 100%) had used it at least once in the 30 days prior to the time of data collection. Among the app users, five individuals used the digital PRISM-S self-assessment of suicidality, six accessed the digital personal safety plan, one used the feature to contact personal acquaintances, two contacted professional support services, two called emergency services, and one used the app notifications. Of those who used the digital PRISM-S self-assessment in the SERO app, 60% (n=3) stated that the digital self-assessment of suicidality is a good alternative to the joint assessment with a professional. One person (20%) disagreed, and another (20%) was unsure. Regarding how users discovered the SERO app, 78% (n=7) of the app users indicated they were made aware of it during their treatment at *lups*. Two other individuals instead provided feedback on the SERO app in the text field. One stated: *“It gives me security”*, while the other reported: *“Unfortunately, it crashes too often, and I had to re-register each time”*. 78% (n=7) of individuals who used the SERO app would recommend it to others, while 22% (n=2) would not.

**Figure 3:**
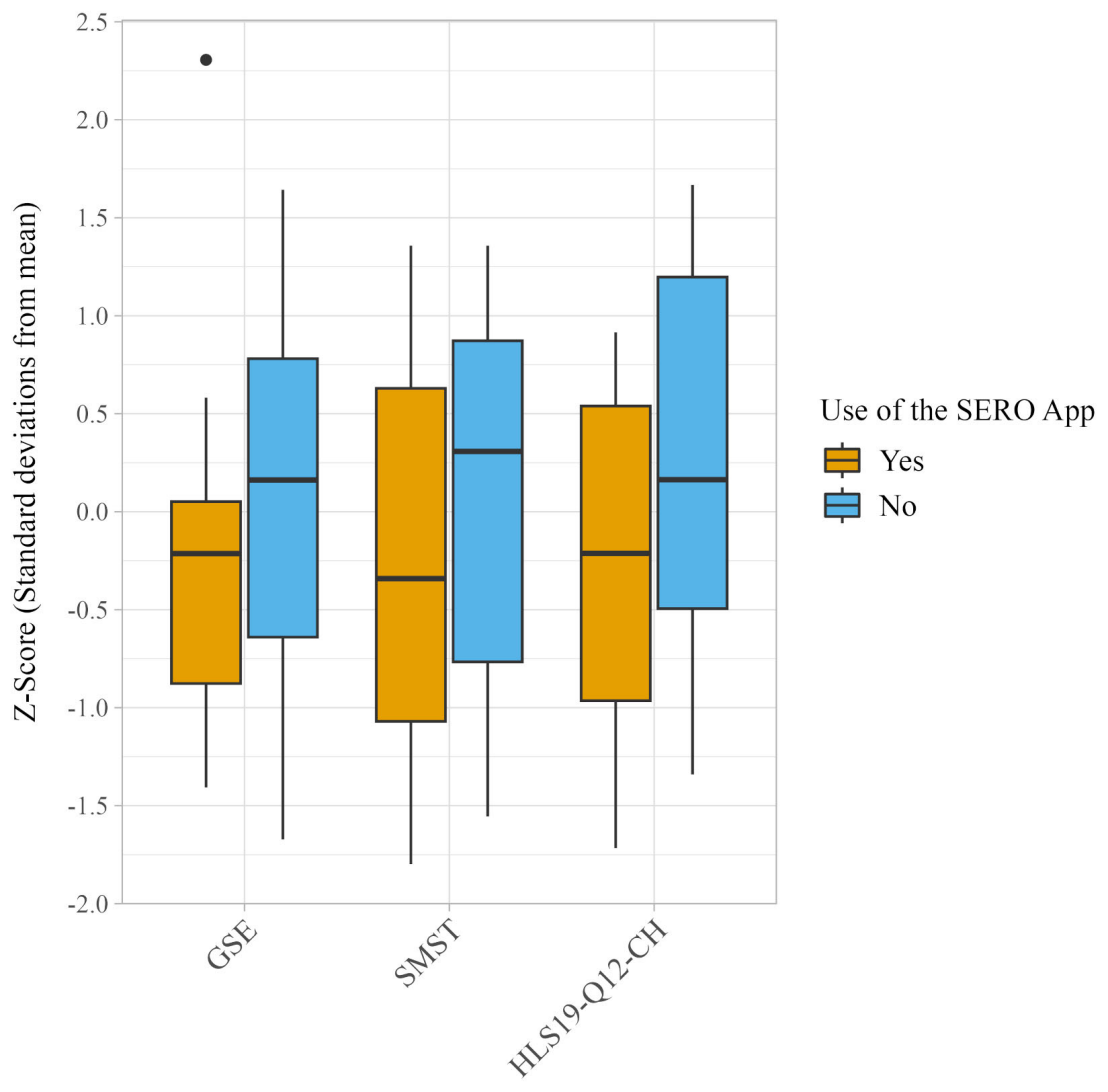
Boxplots depicting the distribution of z-scores on the General Self-Efficacy Scale (GSE), the Self-Management Self-Test (SMST), and the Swiss version of the Health Literacy Questionnaire (HLS19-Q12-CH) categorized by individuals who had and had not used the SERO app. Z-scores represent the number of standard deviations each participant’s score is from the mean score of the group. Higher scores imply better outcomes. No group difference reached statistical significance (GSE: W=51.5, p=0.488; SMST: W=50.5, p=0.447; HLS19-Q12-CH: W=46, p=0.295).

On average, individuals who had used the SERO app at least once reported lower mean scores on the GSE (21.7, SD=8.5), SMST (8.7, SD=4.2), and HLS19-Q12-CH (56.5, SD=19.9), compared to non-users (GSE: 23.2, SD=7.2; SMST: 9.9, SD=4.2; HLS19-Q12-CH: 67.3, SD=23.2). However, none of these differences were statistically significant (GSE: W=51.5, p=0.488; SMST: W=50.5, p=0.447; HLS19-Q12-CH: W=46, p=0.295). **Figure 3** presents a comparative boxplot visualization of mental health outcome scores, standardized as z-scores, between participants who had used the SERO app at least once and non-users.

## 4 Discussion

### 4.1 Mental health in individuals at increased suicide risk after hospital discharge

Study participants had on average poor to moderate scores in self-efficacy, self-management, and health literacy six months after discharge from a psychiatric hospital. This observation aligns with previous research indicating that low levels in these mental health measures are associated with higher levels of suicidal ideation and behavior (19, 22, 42, 43). We observed large differences in responses between individuals and consequently a wide range of scores. For example, participants’ scores spanned the full possible range from the lowest to the highest levels of self-efficacy, indicating significant variability in individuals’ confidence in managing stressful or challenging demands. However, it is important to note that cross-sectional data provide only a snapshot. Thus, the mental health values measured in our study could vary significantly for individuals at different times (44).

The low to moderate levels in self-efficacy, self-management, and health literacy observed in our sample, coupled with their significant relevance to suicidality, underscore the importance of targeted interventions to improve these protective skills in individuals at suicide risk. Furthermore, the diversity in the assessed mental health outcomes points to the need for tailored approaches in health education and support services to cater to varying levels of individual competence. For example, resilience training programs that aim to improve the ability to cope with challenges and overcome crises may be beneficial for managing future suicidal crises (45, 46). To improve mental health literacy, educational programs and workshops that focus on understanding medical and health information, navigating mental health services, and making informed health decisions show promise (47). Our finding that half of our sample demonstrated very poor or rather poor self-management competence underscores the potential benefits of (digital) interventions that provide psychological self-help (48). Digital self-management interventions may be especially beneficial for individuals reluctant or unable to access traditional health services (48). The SERO app is one example of a tool that aims to improve self-management among individuals at increased suicide risk (6).

### 4.2 Usage and usefulness of SERO project components

The study participants were exposed to SERO components to varying degrees, with the PRISM-S technique being the most frequently used component. The PRISM-S method for suicide risk assessment, recommended by the Swiss Federal Office of Public Health (49), has been successfully implemented as the standard for suicide risk assessment in the treatment settings of *lups*. The development and use of the personal safety plan, usage of the SERO app, and participation in ensa courses are at the discretion of at-risk individuals or their relatives. Subject to the small sample size of our study, the usage rates of 67% for the safety plan and 38% for the SERO app suggest that these tools are well-received by many members of the target group. The uptake of the ensa courses by relatives of study participants was low, suggesting that additional measures may be necessary to improve awareness, acceptance, and utilization of this resource. Marketing strategies might need to be reconsidered or access to the courses increased to encourage broader usage.

Besides personal choices for or against specific SERO components, it must be considered that not all components were fully implemented in every *lups* ward and were accessible to or known by all patients at the time of some participants’ discharge. For example, the SERO app has been available since December 2022. Although we started collecting data in March 2023, the first study participants could not have been aware of the SERO app during their *lups* treatment six months earlier. Subsequent research should re-examine the usage of SERO’s components now that they are fully integrated and accessible.

More than half of the participants (63%) who developed a personal safety plan at *lups* used it in the 30 days prior to data collection. This suggests that the safety plan remains relevant to these individuals at suicide risk six months after hospital discharge. Safety planning aims to prepare individuals for future suicidal crises (15) and is recognized as a good-practice measure in SP (14). 50% of study participants who developed a personal safety plan indicated that this tool has helped, or could help, before or during a suicidal crisis. This finding aligns with previous research, which demonstrated that safety planning interventions are associated with improvements in suicidal ideation and behavior, reductions in depression and hopelessness, and fewer hospitalizations (50). The differences in how well the SERO safety plan meets individual needs should be explored in more detail, for example through qualitative interviews, to further refine the instrument. The relatively balanced distribution of individuals who used the safety plan on paper, in the SERO app, or both, supports the recommendation by Duke et al. (51) that resources for suicide risk safety planning should be available in different formats.

The SERO app’s perceived usefulness provides insights into the potential of digital self-management tools to support individuals at suicide risk in managing their suicidality. 44% of app users provided positive feedback on its usefulness before or during a suicidal crisis, 78% would recommend the SERO app to others, and all used it at least once in the 30 days prior to data collection. These findings suggest that the SERO app addresses existing needs and could be a valuable support for many individuals at suicide risk. Although current evidence on the effectiveness of digital SP interventions in reducing suicidal behavior is limited, these tools may help manage suicidal thoughts (52) and reduce suicidal ideation (53). The SERO app provides easy and immediate access to a suicide risk self-assessment, contributes to the empowerment and autonomy of individuals in managing their mental health, and could easily be scaled up (52). The free availability of the app in German, French, Italian, and English for iOS and Android devices (7) maximizes the potential reach and use of its features. However, research projects investigating the causal effects of the SERO app on users’ mental health and suicide outcomes, and comprehensive cost-effectiveness analyses are still pending. A detailed examination of user experiences, including addressing technical issues, would support the iterative improvement of the SERO app.

We observed lower scores in self-efficacy, self-management, and health literacy among individuals who used the safety plan or the SERO app compared to non-users. Although these differences were not statistically significant, they raise the hypothesis that people with poorer skills in these mental health domains and potentially greater mental distress may be more inclined to seek additional support services, such as the safety plan or the SERO app. Previous studies have shown a positive correlation between more severe functional impairments and increased help-seeking for mental health problems, indicating that some individuals may seek help only when mental health issues worsen (54, 55). We recommend examining the motivation for using the safety plan and the SERO app, along with the clinical differences between users and non-users, to investigate our hypothesis. Furthermore, future research should assess the causal relationships between SERO’s components and mental health outcomes, including suicide risk, in a larger-scale study. The intertwining of some project components through the SERO app, which includes the digital versions of the PRISM-S self-assessment and the personal safety plan, should be considered in the research design. To compare the value of the project components with other comparable SP measures, a cost-effectiveness analysis should be carried out. With the planned availability of the SERO app in Germany, alongside its current use in Switzerland, there is potential for research projects with a large study population from two countries.

### 4.3 Limitations

The main limitation of our study is its small sample size, which stems from our focus on patients discharged from the psychiatric hospital in Lucerne (*lups*), the inclusion criteria regarding suicide risk and age, and a low response rate. The findings must therefore be interpreted with caution and should be regarded as initial insights into the usefulness of the SERO components rather than a comprehensive assessment of the project. Furthermore, there is potential for selection bias because the individuals’ decision to use SERO components and to participate in our research project might not have been random. For example, individuals who agreed to participate in our study may have felt fundamentally better (or worse) than those who chose not to complete the questionnaire. Additionally, the fact that we did not control for mental health status at discharge, combined with the significantly higher proportion of female participants (83%), limits the representativeness of our study results for individuals at suicide risk. Another issue to consider is that the time of recruitment (six months after hospital discharge) may not necessarily be the last hospital discharge before study participation for all approached individuals. The fact that some participants underwent a subsequent rehospitalization and discharge within the six-month period post-discharge limits the comparability of the questionnaire results. Given that mental health status can change rapidly, particularly among individuals with mental instability and increased suicide risk (44), the timing of questionnaire completion can have a considerable influence on the assessed mental health outcomes. Suicidality is strongly associated with diverse risk factors, including mental disorders, a history of self-harm, adverse life events (56), specific personality traits (57), personality disorders, and drug and alcohol use (58), which we did not control for in our analysis. Given the potential confounding biases, we refrain from drawing any causal conclusions from the data presented. Also, as already noted, the indicated usage rate for the SERO app should be seen as an approximation, since participants discharged before December 2022 did not have the opportunity to use the app, at least not immediately following their discharge. Due to the study materials being solely in German, only German speakers could participate in the study. Despite our study’s focus on the German-speaking cantons of Lucerne, Obwalden, and Nidwalden, its representativeness for individuals at suicide risk in this region is further limited due to Switzerland’s demographic diversity and high level of immigration (59).

## 5 Conclusion

Low to moderate levels of self-efficacy, self-management, and health literacy six months after discharge from a psychiatric hospital underscore the need for targeted psychosocial measures and public health interventions aimed at supporting individuals at suicide risk. This study is the first scientific investigation into the components of the Swiss SP project SERO, providing important insights into their usage and usefulness. Positive feedback on the personal safety plan and the SERO app suggests their potential effectiveness in supporting at-risk individuals during the vulnerable transition from inpatient to outpatient care and beyond. Therefore, incorporating structured and integrated measures for promoting self-management into standard care protocols in psychiatric hospitals and into patients’ lives may contribute to preventing suicides.

Further validation of our findings in a subsequent, larger-scale study with a controlled research design is necessary. To increase the response rate, study materials should also be provided in French, Italian, and English, in addition to German. Furthermore, alternative or complementary recruitment and participation methods could be employed. For instance, conducting data collection earlier than six months post-discharge could capture more recent experiences with SERO. Direct referrals to the evaluation study by healthcare professionals at *lups*, coupled with incentives for study participation, could further enhance response rates. Future research should comprehensively evaluate the project components, analyze user experiences, assess causal relationships with mental health and suicide outcomes, and estimate the cost-effectiveness of each component separately and in combination. This will help ensure that the most effective and economically efficient measures for suicide prevention are scaled up and sustained.

## Data Availability

The original contributions presented in the study are publicly available. This data can be found here: https://doi.org/10.5281/zenodo.10985540.

## Members of the SERO Study Group

Oliver Bilke-Hentsch, Martin Fluder, Johann Meinhof, Kerstin Gabriel Felleiter, Stefan Büchi, and Jochen Mutschler.
